# Comparison of Fluorometric and UV Spectrophotometric Findings for DNA Isolated From Formalin-Fixed Paraffin-Embedded Blocks, Fine Needle Aspiration Cytology Smears, and Blood

**DOI:** 10.7759/cureus.19583

**Published:** 2021-11-15

**Authors:** Pranoy Paul, Swati Rajput, Prashant Joshi, Manisha Naithani, Nilotpal Chowdhury, Shalinee Rao, Manju O Pai

**Affiliations:** 1 Division of Molecular Biology, Proteomics and Metabolomics, All India Institute of Medical Sciences, Rishikesh, IND; 2 Department of Pathology and Laboratory Medicine, All India Institute of Medical Sciences, Rishikesh, IND

**Keywords:** fluorometer, uv spectrophotometer, ffpe, blood, fnac, dna

## Abstract

Introduction: Fine needle aspiration cytology (FNAC) smear may serve as a convenient sample for DNA extraction for molecular pathology in addition to more commonly used formalin-fixed paraffin-embedded (FFPE) sections. DNA quantification done by fluorometer is more accurate than UV vis spectrophotometer regardless of the source. This study was conducted to compare DNA yield and quality from cytology smears, FFPE sections and peripheral blood using both fluorometer and spectrophotometer. Further, introspection was made to check for the adequacy of DNA extracted from cytology smears with respect to DNA extracted from core biopsies.

Method: DNA was extracted from 10 fresh peripheral blood samples, core biopsies and FNAC smears. The DNA was quantified using a fluorimeter and UV vis spectrophotometer in all cases.

Results: Statistically significant difference was seen between the data obtained from UV vis spectrophotometry and flourometry. The quantity of DNA extracted from FNAC smears was higher than that of core biopsy as per fluorometry data (mean DNA of core biopsy = 1.9ng/µl, of FNAC = 3.3ng/µl).

Conclusion: DNA estimation by fluorometry is more accurate and precise than spectrophotometry in FFPE, FNAC and whole blood samples. DNA yield from FNAC slides is comparable to that from core biopsies.

## Introduction

Formalin-fixed paraffin-embedded (FFPE) tissue has been the cornerstone of histopathological examination. As per the evidence in literature, Goelz et al. first described a method to isolate double-stranded DNA from archived FFPE blocks in 1985 [1]. Archival cytology smears obtained from fine needle aspiration cytology technique (FNAC) can be another appropriate source for isolating nucleic acids for downstream processes [2-4]. The quality of DNA extracted from these samples is significantly compromised due to fragmentation and chemical modifications in the extracted DNA [5,6]. In contrast, DNA extracted from fresh blood has relatively higher DNA yield and shows less fragmentation with increased amount of longer DNA fragments [7].

The most commonly used method for nucleic acid estimation in reference labs is UV vis spectrophotometer which relies on the absorbance of light by nucleic acids at different wavelengths [8]. A simplified and portable but less commonly used method of estimating nucleic acid concentration is by fluorometer. According to numerous independent studies, fluorometer is shown to be a much more accurate and reproducible mode of nucleic acid estimation [9,10]. UV vis spectrometer is known to over-estimate DNA or RNA quantity by manifolds [5,10]. Since most mutation analysis kits available in the market do not explicitly mention mode of DNA estimation in their methodology for preparation of amplification mix, using UV vis spectrophotometer readings to set up a reaction may severely decrease the sensitivity of the assay.

To this effect we compared DNA yield and their quality from cytology smears, FFPE sections and peripheral blood using both fluorometer and spectrophotometer. Another objective of the study was to assess the adequacy of scrapings from archived FNAC slides vis a vis FFPE blocks with respect to DNA yield.

## Materials and methods

This study was conducted in the Division of Molecular Diagnostics in collaboration with the Department of Pathology, All India Institute of Medical Sciences, Rishikesh. Mutation analysis for EGFR and KRAS is routinely done in the laboratory using FFPE blocks. In cases where biopsy is unavailable, cytology samples in the form of cell blocks and smears are utilized to extract DNA. We routinely receive peripheral blood samples for detection of HLAB-27 mutations. For this research study, we chose 10 routine samples each from FFPE blocks, cytology smears and peripheral blood whose DNA was isolated in the period between April 2020 to April 2021 for routine testing.

DNA extraction from FFPE blocks

FFPE blocks were prepared from tru-cut biopsies of lung carcinomas. Histopathology slides were screened and tumour area was marked. Blocks having more than 20% tumour area were selected and three to four shavings of 10-micron thickness were taken for DNA extraction. Extraction was done using QIAamp DNA FFPE tissue kit (Qiagen, Hilden, Germany). DNA was eluted in 40µl elution buffer supplied by the manufacturer.

DNA extraction from stained cytology smears

After establishing the diagnosis of non-small cell lung carcinoma on smears, slides having the highest cellularity were selected. In cases where cellularity was low (<500 cells) multiple stained smears were taken and minimum cellularity was established at ~500 cells per case. Alcohol-fixed Papanicolaou (PAP) stained smears were preferred over air-dried smears stained with May Grunwald Giemsa (MGG) stain, however in three cases MGG stained slides were chosen due to higher cellular count. The slides were dipped in xylene bath for a minimum of 48 hours at 56ºCelsius or till their cover slips slipped off. The slides were decolourised by dipping into 0.5% acid-alcohol solution for 30 seconds. Slide scrapings were taken from each of the slides in a microcentrifuge tube. DNA extraction was done using QIAamp FFPE DNA extraction kit (Qiagen) using the manufacturer’s protocol with minor modifications. The modifications made were as follows: addition of xylene for deparaffinisation in the microcentrifuge tube was avoided, and incubation was only done for two to four hours at 56ºCelsius with proteinase K. DNA was eluted in 40µl elution buffer supplied by the manufacturer.

DNA extraction from whole blood

DNA was extracted from the peripheral blood using DNeasy Blood and Tissue kit (Qiagen) using 200µl whole blood as per the manufacturer’s instruction. DNA was eluted in 100µl elution buffer.

DNA estimation

DNA quantity was estimated using Quantus Fluorometer kit (Promega, Madison, WI, United States) for further downstream reactions. Eluted DNA was also measured using QIAxpert UV/VIS spectrophotometer (Qiagen). DNA quality was estimated using 260/280 ratios.

DNA gel electrophoresis

Gel electrophoresis was done for all the extracted samples on 1.5% agarose gel at 150V for 30 minutes using 8µl DNA samples mixed with 2µl bromophenol blue dye. For reference a 100 base pair DNA ladder was used. The gel was visualized under UV light using Azure Biosystems c300 Gel documentation system (Azure Biosystems, Dublin, CA, USA).

## Results

**Figure 1:**
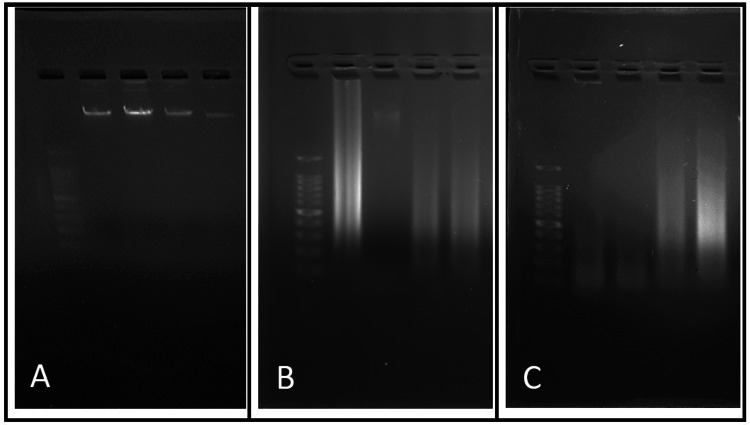
Gel electrophoresis done on 1.5% agar with DNA extracted from A) Whole blood, B) Cytology smears C) FFPE blocks Well 1 (A,B,C) - 100bp ladder FFPE- formalin-fixed paraffin-embedded

Mean DNA yield in blood, FFPE and FNA cytological smear measured by fluorometer and spectrophotometer was found to be 10.99, 1.9, 3.3 (ng/µl) and 29.76, 69.9, 119.9 (ng/µl) respectively (Tables 1, 2, 3). Blood samples showed relatively lesser variation compared to FFPE and FNAC samples. Average total yield of DNA in blood, FFPE and FNA cytological smear measured by fluorometer and spectrophotometer was found to be 1099, 76.52, 132.3 (ng) and 2976, 2797, 4758.7 (ng) respectively. A representative gel electrophoresis picture (1.5% agarose) showed relatively intact DNA in blood samples, whereas both FNAC and FFPE samples showed smeared patterns suggestive of fragmented DNA (Figure 1).

**Table 1:** DNA values from haematology smears TLC- Total Leukocyte Count, S- Spectrophotometer, F- Fluorometer

Case	TLC (x10^3^/µl)	DNA (F) (ng/µl)	DNA (S) (ng/µl)	260/280	Difference (S- F)	Total yield (F) (ng)	Total yield (S) (ng)
Blood 1	9.4	23	43.5	2.2	20.5	2300	4350
Blood 2	4.2	6.3	24.9	2.16	18.6	630	2490
Blood 3	5.1	7.7	22.4	2.57	14.7	770	2240
Blood 4	7.4	11	38.3	2.14	27.3	1100	3830
Blood 5	4.9	10	32.4	1.99	22.4	1000	3240
Blood 6	4.9	7.5	35.21	1.98	27.71	750	3521
Blood 7	5.6	15	20.7	1.88	5.7	1500	2070
Blood 8	4.1	4.6	23.2	1.8	18.6	460	2320
Blood 9	5.2	6.8	21.25	2.1	14.45	680	2125
Blood 10	8.6	18	35.8	1.85	17.8	1800	3580

**Table 2:** DNA values from FFPE blocks S- Spectrophotometer, F- Fluorometer, FFPE- formalin-fixed paraffin-embedded

Case	Cellularity (approx.)	DNA (F) (ng/µl)	DNA (S) (ng/µl)	260/280 ratio	Difference (S-F)	Total yield (F) (ng)	Total yield (S) (ng)
FFPE 1	1000	0.45	37.5	2.17	37.05	18	1500
FFPE 2	1000	4.9	95.7	1.82	90.8	196	3828
FFPE 3	800	1.95	35.6	1.97	33.65	78	1424
FFPE 4	1000	0.192	229	1.93	228.8	7.68	9160
FFPE 5	2000	0.613	38.1	1.82	37.49	24.52	1524
FFPE 6	1000	0.155	19.4	2.18	19.25	6.2	776
FFPE 7	1000	2.35	15.1	3.02	12.75	94	604
FFPE 8	700	0.29	34.1	1.97	33.81	11.6	1364
FFPE 9	2000	3.6	124	2.24	120.4	144	4960
FFPE 10	1000	4.63	70.8	1.88	66.17	185.2	2832

**Table 3:** DNA values from cytology smears S- Spectrophotometer, F- Fluorometer

Case	Cellularity (approx.)	DNA (F) (ng/µl)	DNA (S) (ng/µl)	260/280 ratio	Difference (S-F)	Total yield (F) (ng)	Total yield (S) (ng)
Cyto1	2000	1.21	31	2.54	29.79	48.4	1243
Cyto2	1000	2.71	55.03	1.69	52.32	108.4	2201
Cyto3	5000	1.43	158	2.14	156.57	57.2	6320
Cyto4	700	0.794	46.7	1.78	45.90	31.76	1868
Cyto5	1500	2.82	622	1.88	619.18	112.8	24880
Cyto6	1000	1.34	26.4	1.97	25.06	53.6	1056
Cyto7	1500	3.29	17.2	2.26	13.91	131.6	689.6
Cyto8	1500	3.12	18.4	3.23	15.28	124.8	736
Cyto9	5000	16	195	1.95	179	640	7800
Cyto10	1000	0.355	19.83	1.64	19.475	14.2	793.2

Wilcoxin signed ranked tests were applied on the values obtained from the spectrophotometer and fluorometer. Statistically significant difference was noted between the values obtained from all the three sample types using the two different modalities of DNA estimation (Table 4).

**Table 4:** DNA assay values from blood, core biopsies and FNAC samples. P-Value is obtained from Wilcoxin signed rank test between the Fluorometer and Spectrophotometer values. S- Spectrophotometer, F- Fluorometer, FNAC- fine needle aspiration cytology, FFPE- formalin-fixed paraffin-embedded

	Fluorometer (ng/µl)			Spectrophotometer (ng/µl)			Difference (ng/µl) (S – F)			P-Value
	Mean	Range	Median	Mean	Range	Median	Mean	Range	Median	
Blood	10.99	4.6 -23.0	8.85	29.76	20.7 - 46.9	28.65	18.78	5.7- 27.7	18.6	0.006
FFPE	1.9	0.0.15-4.9	1.28	69.9	15.1 – 229	37.8	68.01	12.75 - 228.8	37.27	0.002
FNA	3.3	0.36 -16	2.07	119.9	17.2 – 622	38.85	115.65	13.9 - 619.18	37.85	0.002

## Discussion

In molecular diagnostics, a major and critical step that influences the accuracy of test is availability of high quality genomic DNA [11]. Quality and quantity of DNA both have an impact on polymerase chain reaction (PCR) efficiency. Therefore, it is also essential to evaluate and compare the techniques for DNA assay. As evident from the results, both core biopsies and FNA samples have comparative DNA yields when measured using fluorometer. While estimating DNA quantity using spectrophotometer, the results were widely fluctuating with average DNA yield by spectrophotometer and fluorometer ratio (qS/qF) of 36.55 and 35.97 respectively for FFPE and FNAC samples. In the fresh blood samples, the qS/qF ratio was found to be 2.71. These findings were in concordance with similar studies conducted by Kumar et al., Deben et al. and O’Neill et al. [5,10,12].

In a fluorometer, nucleic acids are quantified using highly sensitive and accurate fluorescent dyes. Separate dyes are used for double-stranded (ds) DNA, single-stranded (ss) DNA or RNA estimation which increases its specificity. Binding of the dye to dsDNA emits fluorescence at a specific wavelength which is then estimated by the fluorometer and the DNA is quantified. This eliminates the possibility of contamination by RNA, free nucleotides and other proteins. Nakayama et al. demonstrated a good correlation between FFPE-DNA estimated using fluorometer and quantitative (q) PCR, while DNA was significantly overestimated by spectrophotometer [9]. They also postulated that while qPCR may be the most accurate method to estimate DNA quantity and purity, it is very expensive and impractical for routine use and fluorometers offer a cheaper and relatively accurate alternative.

Spectrophotometers use the principle of light absorbance at 260nm by the nucleic acids. In general, it doesn’t distinguish between dsDNA, RNA, proteins or free nucleotides in the sample leading to potentially overestimation of DNA content. When extracting DNA from processed samples like FFPE and FNA smears, it has to be borne in mind that the tissue is subjected to numerous physical and chemical agents which leads to fragmentation of the DNA and potentially increasing the chance of contamination by chemicals. Modification is introduced by chemicals results in protein cross-links between protein and DNA, deamination and adduct formation [13]. Consequence of these modifications result in deterioration of quality and number of amplifiable DNA templates which has a significant effect on PCR sensitivity and specificity [14]. In contrast, fresh blood has relatively preserved DNA along with fewer contaminants. This hypothesis was amply evidenced by our test findings which showed significant differences when using the two methods of DNA estimation.

On comparison of DNA yield from scraped smears and FFPE section it was seen that the average yield of DNA from a single scraped smear with adequate cellularity was greater than 3 x 10-micron sections from FFPE. FFPE sections showed the least correlation between fluorometric and UV spectrophotometric data. FNAC smears yield whole nuclei and do not undergo extensive processing and fixation unlike FFPE sections, and hence may be considered a preferred alternative to FFPE sections or cell blocks [15]. One major drawback in using FNAC smears is that the slides are destroyed while extracting DNA, and hence in cases having limited slides, photomicrographs need to be taken of the representative areas before extraction. In a study by Hartley et al. they demonstrated that DNA yield per nuclear area is better in FNAC smears and FFPE samples require more DNA quantity to achieve comparable mutation detection rates [16].

## Conclusions

Spectrophotometry-based estimation of DNA is highly inaccurate. This inaccuracy increases when using DNA extracted from FFPE and FNAC specimens, but is also less accurate for DNA extracted from fresh blood. Fluorometry-based DNA estimation is more accurate and precise than spectrophotometry in FFPE, FNAC and whole blood samples. Fluorometry-based estimation should always be preferred over spectrophotometer results for quantification of DNA for downstream studies especially when working on core biopsies, FNAC smears or tissue specimens as demonstrated in the study. The present study also confirmed that average DNA yield from FNAC smears was superior or equivalent to DNA yield from FFPE sections and should be considered as a suitable alternative in molecular testing. 
